# Analogies can speed up the motor learning process

**DOI:** 10.1038/s41598-020-63999-1

**Published:** 2020-04-24

**Authors:** Oryan Zacks, Jason Friedman

**Affiliations:** 1 Sagol school of Neuroscience, Tel Aviv University, Tel Aviv, Israel; 2 Dept. of Physical Therapy, Stanley Steyer School of Health Professions, Sackler Faculty of Medicine, Tel Aviv University, Tel Aviv, Israel

**Keywords:** Motor control, Neuroscience, Learning and memory

## Abstract

Analogies have been shown to improve motor learning in various tasks and settings. In this study we tested whether applying analogies can shorten the motor learning process and induce insight and skill improvement in tasks that usually demand many hours of practice. Kinematic measures were used to quantify participant’s skill and learning dynamics. For this purpose, we used a drawing task, in which subjects drew lines to connect dots, and a mirror game, in which subjects tracked a moving stimulus. After establishing a baseline, subjects were given an analogy, explicit instructions or no further instruction. We compared their improvement in skill (quantified by coarticulation or smoothness), accuracy and movement duration. Subjects in the analogy and explicit groups improved their coarticulation in the target task, while significant differences were found in the mirror game only at a slow movement frequency between analogy and controls.

We conclude that a verbal analogy can be a useful tool for rapidly changing motor kinematics and movement strategy in some circumstances, although in the tasks selected it did not produce better performance in most measurements than explicit guidance. Furthermore, we observed that different movement facets may improve independently from others, and may be selectively affected by verbal instructions. These results suggest an important role for the type of instruction in motor learning.

## Introduction

Using analogies in the acquisition of a motor skill has been shown to improve motor learning in various tasks and settings, from throwing a basketball to performing a high jump, and in populations ranging from young children to older adults^1–4^. Analogies in the case of motor learning combine various task-relevant rules into a single biomechanical metaphor, usually given to the learner as a verbal instruction. A survey study among researchers and practitioners^5 ^defined analogy learning as follows: “Learning facilitated by metaphors. The complex structure of the to-be-learned skill is integrated into a simple metaphor that the learner is provided with”. Two examples of this method are given: “reach for an apple up in the tree” (for a jumping pattern) and “putting your hand into the cookie jar” (for a basketball shot). The use of analogies in motor learning has been shown to be beneficial both during the learning process and in maintaining performance under pressure^2,6^.

Most of the research in this field has been done under the implicit/explicit knowledge paradigm. Researchers have defined explicit motor learning as conscious control of a motor task. This type of knowledge structure relies on working memory and is characterized by learning rules that govern the movement and being able to explicitly state such rules. In contrast, implicit knowledge of a motor task doesn’t necessitate conscious awareness of specific rules and isn’t as demanding on working memory^7^.

Experiments show that explicit knowledge can hinder performance during the learning process, although this occurs mostly under stressful conditions^8^. By implicitly learning, participants can become more efficient in a motor task without being explicitly told how to improve^9^. Implicit learning techniques can involve the use of a secondary cognitive task designed to occupy working memory and may reduce the chance that learners consciously form explicit rules or test hypotheses about their performance of the task^10^. While such studies have demonstrated the advantages of implicit learning, the methods used in these studies aren’t necessarily easy to apply in the real world.

Another lens from which to view the use of analogies in motor control is the placement of attention during the task. There is a vast literature concerned with conscious monitoring and focus in the execution of skilled tasks and its negative effects on performance under pressure, referred to as “choking”. Jackson, Ashford & Norsworthy^11 ^showed that while dual tasks can contribute to performance, focusing on the skill itself or goals related to the motor task at hand can hinder performance, especially under stress. Furthermore, recent research reveals that internal focus and conscious control hinder performance in motor learning and execution. External focus, however, was shown to improve performance^12^. Using analogies can aid in directing attention to external features of the movement or to its results^13^.

In this study we aimed to apply this interesting technique to motor control, investigating its effect on movement primitives and strategy preference. We tested how the use of analogies influences motor kinematics as well as task outcomes. We hope this study will open an additional perspective on the ways in which cognitive processes such as attention, consciousness and imagination influence motor control and output.

We chose to focus on kinematic aspects of motor skills that have been under-represented in the literature on analogy learning surveyed thus far. One kinematic measure of skilled movement is smoothness, defined here as a continuous movement without interruptions^14^. Smoothness is a marker of healthy and skilled movement and is used to asses motor recovery after stroke^15^, and motor learning in healthy adults. It can also be linked to effort minimization and spatial and temporal coordination, as well as a reflection of the cognitive representation subjects might have of a certain movement^16^.

The first task we used was a sequence of planar hand trajectories passing through several targets, in which participants can employ different strategies in order to complete the task more quickly and smoothly. The protocol is similar to the one used in Sosnik *et al*.^16^ and Friedman & Korman^17^, a point-to-point drawing task, in which participants are asked to draw lines connecting 4 dots in a specified order (ABCDA).

Previous experiments with this task have shown participants tend to begin with a “fragmented strategy”, in which they draw 4 discrete lines, one for each connection between the dots. Over many practice trials, most subjects “smooth out” their movement, arriving at two curved lines. With no explicit instructions, this transition takes several days (and hundreds of repetitions) to occur. In a previous study, we observed that demonstration by a model can speed up the learning process^17^. In the current experiment, we tested the hypothesis that motor strategy can change when participants are given an appropriate verbal analogy, without explicit instructions or demonstrations on how to perform the task. We compared the performance of the group receiving the analogy instructions with a control group that didn’t receive any further instructions, and a group that received explicit instructions on how to perform the task.

The second task is meant to test if the use of analogies can help in motor performance at the limits of the human motor repertoire. Previous studies have shown that humans aren’t able to perform slow (low frequency) movements in a smooth fashion, generating “jitter” (i.e., unnecessary acceleration zero crossings) throughout a tracking task^18^named “the mirror game”. We used a protocol similar to that from Noy *et al*.^18^, where participants tracked a moving oval with a hand-held stylus on a tablet.

Our main question is whether analogies improve performance in tasks that would demand many hours of practice without such instructions. We hypothesized that hearing a verbal analogy would allow participants to quickly achieve skillful movement, relative to performance of a control group, who received no instructions regarding the strategy to use, or a group that received explicit instructions regarding strategy for performing the task. In the current study we didn’t apply stressful conditions in testing performance. Many analogy studies use such methods to show the superiority of implicit learning. Here we were interested in providing insights that would lead to new strategies for motor learning. For this reason, we expected the analogy group to perform as well or better than the explicit group in both tasks.

In the target task specifically, we expected participants of the analogy and control groups to improve shortly after the additional instructions were given and then maintain the skillful movement throughout the experiment with little additional gains^19^. Regarding the mirror game, we expected the analogy and explicit groups to reduce the amount of jitter generated in their movement after receiving the instruction, while the control group would maintain their baseline performance.

## Results

### Target task

**Figure 1:**
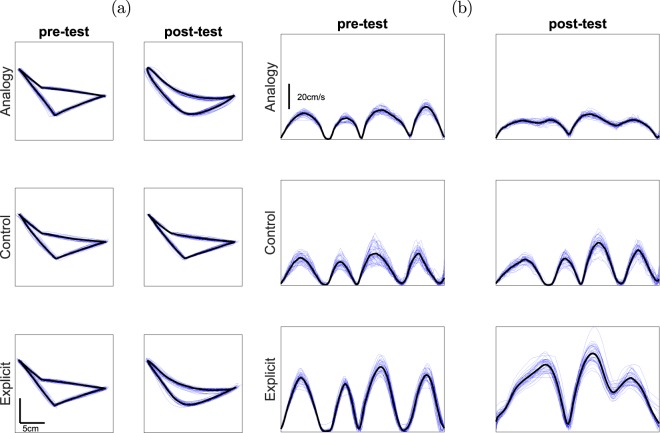
Combined trial paths and tangential velocities of representative subjects. (**a**) Graphs of all trajectories (blue lines) and mean trajectory (black line) from a representative subject from the three groups, from the pre-test and post-test. The x axis corresponds to horizontal movements (left-right) and the y axis corresponds to forward and back movements. All graphs have the same scale. After hearing the analogy and in the explicit group, participants were able to draw curved lines, passing through targets B and D without creating sharp angles. (**b**) Tangential velocity profiles following registration (blue lines) and mean tangential velocity (black line) from representative subjects from the three groups, from the pre-test and post-test. The x axis is normalized time, while the y axis is the tangential velocity, all have the same scale. Tangential velocity profiles with troughs approaching zero indicate participants stopped on the targets, thereby separating the path into four distinct movements. Higher troughs indicate a large degree of overlap between movements or coarticulation.

Movement trajectories (Fig. 1(a)) and tangential velocity profiles (Fig. 1(b)) of representative subjects display the baseline and qualitative changes in task performance. At the pre-test phase of the experiment all participants used straight paths to connect the targets (left column in Fig. 1(a)). These were characterized by distinct four-peak tangential velocity profiles with full stops between movements (left column in Fig. 1(b)). After they were given an analogy or an explicit instruction, participants began using curved lines connecting A-B-C and C-D-A targets and they did not fully stop in points B and D (right column in Fig. 1(a,b)). Control group participants maintained approximately the original movement pattern throughout the experiment. Below we present analysis of kinematic components including coarticulation score, number of tangential velocity peaks, spatial error and movement duration.

### Coarticulation score

**Figure 2:**
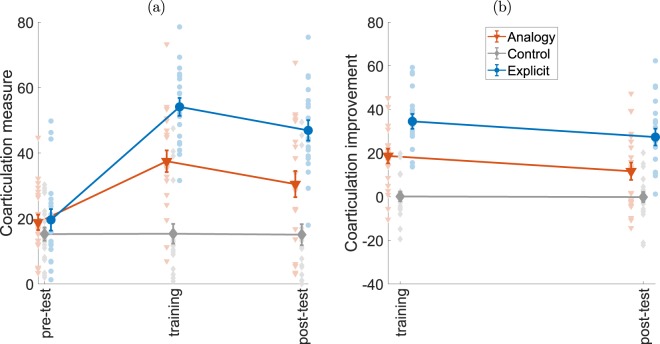
Coarticulation score and improvement. (**a**) Time-course changes in coarticulation score for the three experimental phases. Participants mean coarticulation score was calculated for each phase, shown here as the distribution of individual scores. Bold lines indicate group means, bars indicate SEM. Orange triangles represent the analogy group (n = 21), gray diamonds represent the control group (n = 20) and blue circles represent the explicit group (n = 19). (**b**) Improvement in coarticulation score of each subject compared to their initial performance in the pre-test.

We used coarticulation as the main measure by which to compare groups in this task. The coarticulation score represents progressively higher spatial and temporal overlap of movements and is a sign of skilled performance in this task^16^. Individual and group means of this measure are presented in Fig. 2(a), along with improvement over the experimental phases in Fig. 2(b). The change in coarticulation score differed between the groups, as supported by an interaction of phase and group (F(4,114) = 19, p < 0.0001). Additional main effects were observed for test phase (F(1.6,92) = 58.8, p < 0.0001) and group (F(2,57) = 23.3, p < 0.0001).

Participants in all groups displayed a similar coarticulation score in the pre-test phase (analogy 19 ± 2, control 15 ± 2, explicit 19.5 ± 3). Post hoc t-test analysis did not find a significant difference between groups at the pre-test phase (p > 0.76 in all comparisons), i.e. there were no baseline differences. Furthermore, the control group maintained a constant level of coarticulation throughout the experimental phases (training 15 ± 3, post-test 15 ± 3) and post-hoc t-tests for the phase were not significant for the control group (p > 0.99 in all comparisons).

Participants in the analogy and explicit groups showed a higher degree of coarticulation in the training phase (analogy 37.5 ± 3, explicit 54 ± 3) and in the post-test (analogy 30.5 ± 4, explicit 47 ± 3) compared to the control group (training 15 ± 3, post-test 15 ± 3. training: analogy t(38.8)=4.9, p < 0.0001, explicit t(36.8)=9.5, p < 0.0001; post-test: analogy t(37.6) = 3, p = 0.014, explicit t(37) = 7.1, p < 0.0001). Furthermore, the coarticulation score for the explicit group (training 54 ± 3, post-test 46 ± 3) was higher than the analogy group (training 37 ± 3, post-test 30 ± 4), and this difference was statistically significant in both training (t(37.3) = 3.9, p = 0.0011) and post-test (t(36.86) = 3.1, p = 0.01) phases.

### Number of tangential velocity peaks

This measurement is used as a compliment to the coarticulation score, indicating the number of acceleration periods participants used during their movement. Since the maximum number of peaks did not exceed 4 and thus the data will not be normally distributed, we used the Friedman nonparametric test to compare group means throughout the experiment. While both the analogy and explicit groups improved in the coarticulation score, for the number of peaks in the tangential velocity profile, an improvement (reduction in number of peaks) occurred only for the explicit group. The Friedman test was significant in this measurement (χ^2^(2) = 6, p = 0.0278) and post-hoc analysis revealed a significant difference between the explicit group and the control group (z = 2.449, p = 0.0429), but not between the analogy group and any other group (p > 0.66 for both comparisons). The explicit group mean number of peaks per trial was 3.82 ± 0.07 in the training phase and 3.83 ± 0.07 in the post-test. The analogy group reached 3.94 ± 0.04 in the post-test. All other means were above 3.96. The number of peaks reveals that while participants avoided full stops on the targets (which is contained in the coarticulation score) during their movement, they didn’t fully integrate separate segments into one continuous motion.

### Accuracy and movement duration

**Figure 3:**
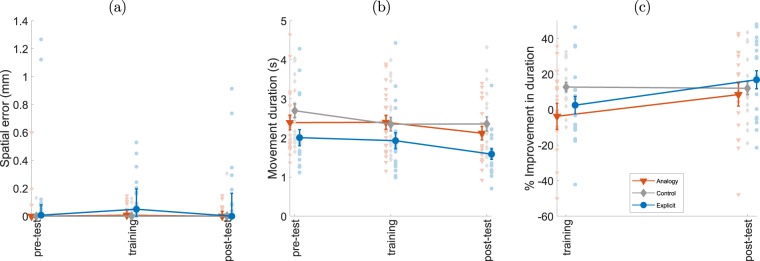
Accuracy and movement duration. (**a**) The median of spatial error per block for individual participants. Bold lines indicate group medians. Spatial error corresponds to distance in mm from subjects’ path to actual target location. (**b**) The median movement duration per block for individual participants. (**c**) The percent of improvement in movement duration, compared to pre-test performance. Bold lines indicate group means, bars indicate SEM. Orange triangles represent the analogy group (n = 21), gray diamonds represent the control group (n = 20) and blue circles represent the explicit group (n = 19).

Spatial error was calculated by measuring participants’ distance from the targets, shown in Fig. 3(a). The error was small throughout the experiment (median less than 0.083 mm for all groups), indicating that all groups maintained a high level of accuracy in all phases. The results of this measure are far from being normally distributed, as many trials contain zero spatial error (i.e., they passed through all the targets). For this reason, we used the Friedman nonparametric test to compare the magnitude of spatial error over the trial phases in each group. This test was not significant for both the analogy group (χ^2^(2) = 2.167, p = 0.34) and the explicit group (χ^2^(2) = 2.98, p = 0.22). The test was significant for the control group (χ^2^(2) = 6, p = 0.0498) but further post-hoc comparisons between test phases were not (p > 0.46 in all tests). We thus infer that while participants in the analogy and explicit groups showed higher coarticulation, they didn’t sacrifice accuracy in order to achieve the different movement pattern.

Movement duration shows yet another pattern of change throughout the experiment (Fig. 3(b)). The explicit group had a lower movement duration in the pre-test compared to the other groups. For this reason, we normalized the results by comparing training and post-test to pre-test for each participant and calculated the percent improvement in movement duration during the experiment (Fig. 3(c)). A main effect was observed for test phase (F(1,114) = 10.17, p < 0.0001) but not for group (F(2,57)=1.126, p = 0.3314) or interaction of phase and group (F(4,114) = 2.148, p = 0.0793). Post hoc tests revealed that the effect was due to the difference between the post-test and the other phases (post-test vs training t(114) = 3.055, p = 0.0084, post-test vs pre-test t(114) = 4.402, p < 0.0001), i.e. movement time was faster in the post-test, but this improvement did not differ across groups.

### Mirror game

**Figure 4:**
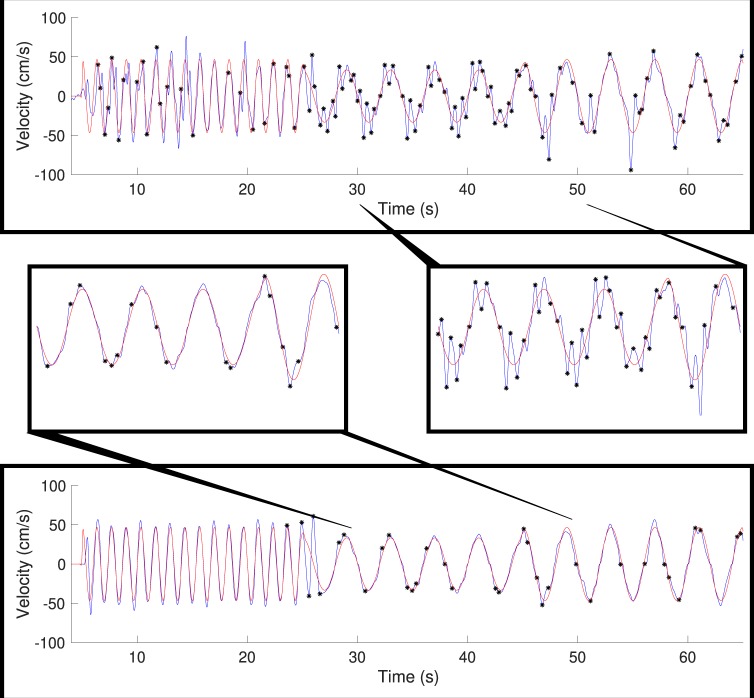
Movement velocity comparison between the groups. Stimuli and responses of representative subjects in low-frequency movements. Images of a full trial completed by a subject of the control group (top) and the analogy group (bottom). Red lines correspond to the stimulus movement velocity across the screen, with the zero-crossings indicating changes in direction. Blue lines correspond with participants’ movement velocity in response to the stimulus. Black stars represent jittery movement, or points of non-obligatory acceleration zero-crossings. Enlargements of specific areas within the trial where jittery movements can be clearly identified are placed in the center.

We recorded synchronization, or tracking ability, in the mirror game at different movement frequencies. Figure 4 displays representative subjects from different groups, in a trial containing both high and low frequencies. The red line in the figure represents the stimulus velocity, which remains smooth throughout the trial, including in transitions between stimulus frequencies. The blue line represents participants’ velocity and is much less regular (as expected). Black asterisks indicate a zero-acceleration crossing of the participant that doesn’t correspond to stimuli direction-changes. We can see qualitative differences in tracking accuracy and the adjustments that appear primarily in low frequency movements. We expected relatively less jitter in the higher frequencies (compared to the lower frequencies) in all groups, as well as an improvement (reduction) in jitter in the analogy and explicit groups.

### Jitter frequency

**Figure 5:**
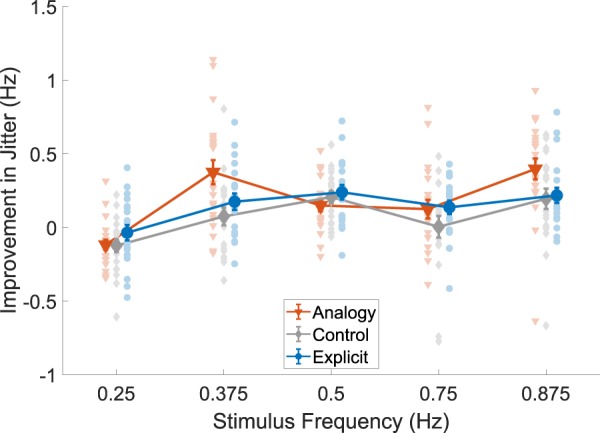
Improvement (reduction) in peak jitter frequency over all stimuli frequencies. Peak jitter frequency was determined as the peak (from the peak of the kernel density plot) of the distribution of jitter (half the reciprocal of the time between distinct points of acceleration-zero crossings). Improvement (i.e., a reduction) in jitter frequency was calculated by subtracting the frequency in the post-test from that of the pre-test. Bold lines indicate group means, bars indicate SEM. Orange triangles represent the analogy group (n = 21), gray diamonds represent the control group (n = 20) and blue circles represent the explicit group (n = 19).

We analyzed the peak frequency of the distributions of jitter events (zero-acceleration crossings) in participants’ movements at different stimuli frequencies across test phases (Fig. 5). In order to determine improvement in performance, we subtracted the baseline mean jitter frequency from the post-test mean for each subject. Due to two missing values in the data set (as a result of recording problems) we used a mixed-effects model (REML) to analyze the results. We found a significant interaction between group and stimulus frequency (F(8,230) = 2.33, p = 0.02), as well as main effects for the group variable (F(2,58) = 4.27, p = 0.019) and stimulus frequency variable (F(3.7,211.5) = 19.6, p < 0.0001). We used Bonferroni multiple comparisons in our post-hoc analysis.

Surprisingly, all groups showed a slight worsening of performance in 0.25 Hz stimulus frequency, the lowest and most challenging frequency (analogy −0.12 ± 0.04 Hz, control −0.12 ± 0.04 Hz, explicit −0.04 ± 0.05 Hz). This was significant compared to all other frequencies (p < 0.008 in all tests). However, we didn’t find significant differences between the groups in this frequency (p > 0.59). For the other frequencies (> = 0.375 Hz), the participants in all groups improved, with the mean improvement for each group (omitting 0.25 Hz) as follows: analogy 0.26 ± 0.07 Hz, control 0.12 ± 0.05 Hz, explicit 0.19 ± 0.02 Hz.

However, in the 0.375 Hz frequency, the analogy group was significantly better (i.e. significantly reduced their amount of jitter by 0.375 Hz from 0.836 ± 0.08 Hz to 0.461 ± 0.02 Hz) compared to the control group (reduced from 0.678 ± 0.06 Hz to 0.604 ± 0.04 Hz; t(36) = 2.95, p = 0.017), although not significantly better than the explicit group (reduced from 0.674 ± 0.05 Hz to 0.499 ± 0.03 Hz; t(35) = 2.02, p = 0.15). All other post-hoc p-values range between p = 0.14 and p > 0.999.

### Tracking accuracy

We analyzed the tracking errors of participants to assess their accuracy in tracking the stimuli. Three measures were used based on Noy *et al*.^18^: relative position error (dX, unitless), relative velocity error (dV, unitless) and mean timing error (dT, in seconds). Improvement in all tracking errors was calculated by subtracting the mean error of each participant in the post-test phase from the pre-test phase and combined to calculate group means. Again, we used a mixed-effects model (REML) to analyze the results.

Stimulus frequency significantly affected relative position error (dX) (F(3.52, 202.5)=22.15, p < 0.0001), although neither group nor the interaction was significant (p = 0.95 and p = 0.38 respectively). Since we are mainly interested in the differences between the groups and their interaction with stimulus frequency, post-hoc tests were not performed. A similar pattern can be observed for relative velocity error (dV), where stimulus frequency significantly affected relative velocity error (dV) (F(2.35,135)=4.58, p = 0.008) but there was no significant effect of group or the interaction (p = 0.4 and p = 0.93 respectively). Post-hoc tests were not performed in this case. The mean timing error (dT) measurement displays a different pattern in tracking accuracy along with higher variability between subjects. In this case the statistical test was not significant for any of the variables (p > 0.4).

## Discussion

We set out to test if analogies can shorten the motor learning process and if their effects can be seen on a kinematic level. In our research we looked at variables such as accuracy, speed and movement skill (as determined by coarticulation or smoothness). As there was no added stress to the tasks in this experiment, and the tasks themselves were simple, we expected the analogy group to perform as well as the explicit group, or better. Our results were less uniform than expected, with the explicit group outperforming the significant gains in coarticulation of the analogy group in the target task. However, the analogy group achieved smoother movements in a low-speed condition of the mirror game.

Coarticulating in the target task can reflect either hours of practice^16 ^or a new insight into the movement pattern^17^. When approaching this task, we anticipated a binary outcome; either participants will gain insight and show faster, coarticulated movement, or they will remain with the initial movement pattern. We were surprised to see that different kinematic aspects display different patterns of improvement (or lack thereof) as well as the significant differences between the analogy and explicit groups. The analogy group displayed a gain of mastery and an improvement in coarticulation, as compared to the control group, although they did not show a concomitant improvement (reduction) in movement time. The explicit group did even better in terms of coarticulation, outperforming the analogy instructions, although they also did not show an improvement in movement time. In both groups, these improvements did not come at the cost of decreased accuracy, i.e., it was not due to changing relative weights in the speed-accuracy tradeoff. Comparing our experiment to the previous results^17^ obtained by observing an expert was also interesting. It seems that the observation groups fell between our analogy and explicit groups, as they weren’t significantly different from either group. However, this shows that verbal instructions can be as potent as observing an expert in some aspects of skill acquisition. Certain verbal instruction, including analogies, can induce insights into motor strategies, although the effects seem to be limited to the kinematic measures most directly related to the instruction or analogy.

The overlap of movements that results in coarticulation may be a step in the direction of forming a new movement primitive^16^. Instead of perceiving the task as composed of four separate movements, participants “chunk” movements together, thinking about the task as composed of 3, 2 or even one movement^20^. We expected the formation of new motor primitives in this task to be reflected in a lower number of velocity peaks. Results indicate that while the analogy group avoided coming to a complete stop on the targets, they did not reduce the number of peaks in the velocity profile. This resulted in “softer” velocity troughs (not reaching zero velocity) but still generated distinct velocity peaks. We interpret these findings as indicating that the analogy was able to capture one aspect of the movement (don’t stop on the targets), but further improved performance by chunking, which would result in fewer velocity peaks, likely requires sleep and consolidation to occur^21^.

As expected, the control group maintained their initial performance throughout the session. While they did have some time to practice, it wasn’t sufficient to gain mastery of the task and generate coarticulated movement. Regarding the duration of movement, we did not see significant differences in improvement between any of the groups throughout the experiment. We attribute the general improvement over the experimental phases to simple practice, since all participants were asked to complete the task as fast and accurately as possible (including before the post-test). Neither the analogy nor the explicit instructions were able to induce an insight into this component of the movement beyond the control group performance. A recent study investigated the possibility of reward increasing motor performance in similar tasks^22^. Reward was able to induce transient increases in speed and even permanent movement chunking. Other research shows that the spatial and temporal aspects of a motor task can be regulated separately, using different feedback cues^23,24^. It is interesting to note that in Friedman & Korman^17^, viewing an expert in full speed caused participants to complete the task much faster (26.7% mean improvement for the observation group).

The mirror game was in some respects a more challenging task, as it wasn’t clear to participants what improvement in it would look like. Although subjects were supplied with visual feedback of their own movement, it’s hard to determine how many corrections one is performing while tracking the stimulus. Overall, participants were not able to achieve better movement performance over the duration of the experiment. Previous research in our lab used this task to show the differential performance of movement under different frequencies^18^. This research indicates that people have a harder time moving smoothly in low frequencies. We attempted to use this to our advantage, and test if we would also see differential improvement at different frequencies.

Analyzing participants’ performance according to the stimulus frequency produced interesting results. To start, all groups showed an increase rather than decrease in jittery movement at 0.25 Hz as the experiment progressed. This is the slowest stimulus provided in the experiment and supposedly the most challenging in terms of smooth movement. It might be that verbal instructions of any kind couldn’t improve participants’ performance, but fatigue impacted this stimulus frequency the most. It may also have been due to differences in the properties of the stimuli between the pre- and post-test phases. Surprisingly, in the next stimulus of 0.375 Hz, all groups showed a decrease of jittery movement, but the analogy group was the only group that significantly improved (compared to the control group). This is still a low-frequency movement, and one that in previous experiments didn’t lend itself easily to smooth movements. Noy *et al*.^18^ considered this frequency below the natural human movement repertoire, so this is a positive indicator in our opinion that analogies can improve such movements.

However, it’s important to note that we expected performance improvement to show a pattern corresponding in some way to the pattern of stimulus frequencies. This wasn’t the case, and other than the analogy group in 0.375 Hz, other interactions weren’t significant. It might be that it is very hard to improve in such a task, regardless of the frequency, or that we did not supply enough practice time or the right feedback to improve. The task itself might also be unsuitable, challenging or not sensitive enough. Studies indicate that performing slow rhythmic motion is hard for humans, due to the organization of the system controlling movement, rather than musculature factors. For this reason, it might be very hard to overcome limitations in this area and show significant improvements^25^. As opposed to the target task, the analogy group performed better than the explicit group at this task in a low-frequency movement. This indicates that explicit instructions have little impact on this type of task. Analogies may help in improving motor skills that are otherwise hard to communicate or implement due to systemic limitations.

We decided to use a similar analogy for both tasks, so we can see the effect it would have in different circumstances. We think that every pair of task and analogy is unique, and they must be aligned in order to have an effect. Borrowing an analogy from one scenario will not necessarily improve results in another. Unfortunately, there has been very little discussion of the content of the analogy in the literature, including why certain analogies are preferred over others. A valuable paper on the subject was published by Poolton, Masters & Maxwell^26^ in which they developed a new analogy that would be more culturally appropriate for Chinese subjects. This was necessary after a previous analogy that improved performance in native-English speakers did not translate well and wasn’t able to aid Chinese learners.

In our study we saw that the analogy can affect separate aspects of the movement differentially, leading to surprising results. For instance, the analogy group’s performance in the target task can be interpreted as a middle point between the control and explicit groups, or a new variation of movements more aligned with their understanding of the instructions. There are certain limitations to kinematic studies. Beyond the limitations mentioned above regarding the mirror game, there may also be floor and ceiling effects present, like the number of peaks measure we used in the target task. Additionally, there’s an inherent challenge in attempting to extrapolate from the planar hand tasks studied here to more complex, full body movement skills. However, we think it could be highly beneficial to incorporate kinematic analysis to future studies of the analogy method and its effects on motor learning and control.

Analogies have been linked to improved performance under stress, reduced cognitive load and improved motor learning^27^. A recent study used EEG recording while participants performed a hockey swing with analogy or explicit instructions^28^. The study demonstrates different neural activity in verbal areas between the two conditions. The authors conclude that using analogies increases cognitive efficacy by decreasing the load of verbal processing. Using both brain-imaging techniques and kinematic analysis will help in developing a better understanding of these cognitive processes.

We would encourage future research to use kinematic analysis of the motor learning process and outcomes. As we have seen, different aspects of movement can be somewhat independent and be differentially affected by instructional methods. Tying such motor outcomes to neurological activity would be interesting and perhaps revealing. Such research would especially benefit rehabilitation practices aimed at patients with both motor deficits and cognitive impairment. It’s important for practitioners to note that the instructions they use can have an impact on patient’s and student’s performance. While some trial-and-error might be needed to find the best match between task and instruction, finding new and creative ways of communicating motor instructions can be worthwhile as a way of enhancing the motor learning process.

## Materials & Methods

### Participants

61 right-handed participants (40 females, average age 25 ± 3) were recruited from the student population at Tel Aviv University. We anticipated that the effect of the analogy will be similar to the effect of viewing an expert^19^, based on an earlier experiment conducted in our lab. In order to show a significant difference between the group with the intervention (coarticulation score:25 ± 17), compared to the control group (coarticulation score: −4 ± 11) with a power of 0.95, we require 14 participants per group. We recruited 20 participants per group to overcome dropouts and data recording problems.

Right handedness was confirmed using the Edinburgh inventory^29^. All subjects were randomly assigned to begin with either the mirror game or the target task, and instructions were counterbalanced between analogy, explicit and control (no instruction). In each task there were 21 subjects who received an analogy, 20 assigned to the explicit group and 20 assigned to the control group. The experiment design was ethically approved by the Tel Aviv University Institutional Review Board, and the study was performed in accordance with the relevant guidelines and regulations. Participants signed an informed consent form before beginning the experiment and were paid for their participation (50 NIS, equal to approximately $13.5).

### Experimental setup

**Figure 6:**
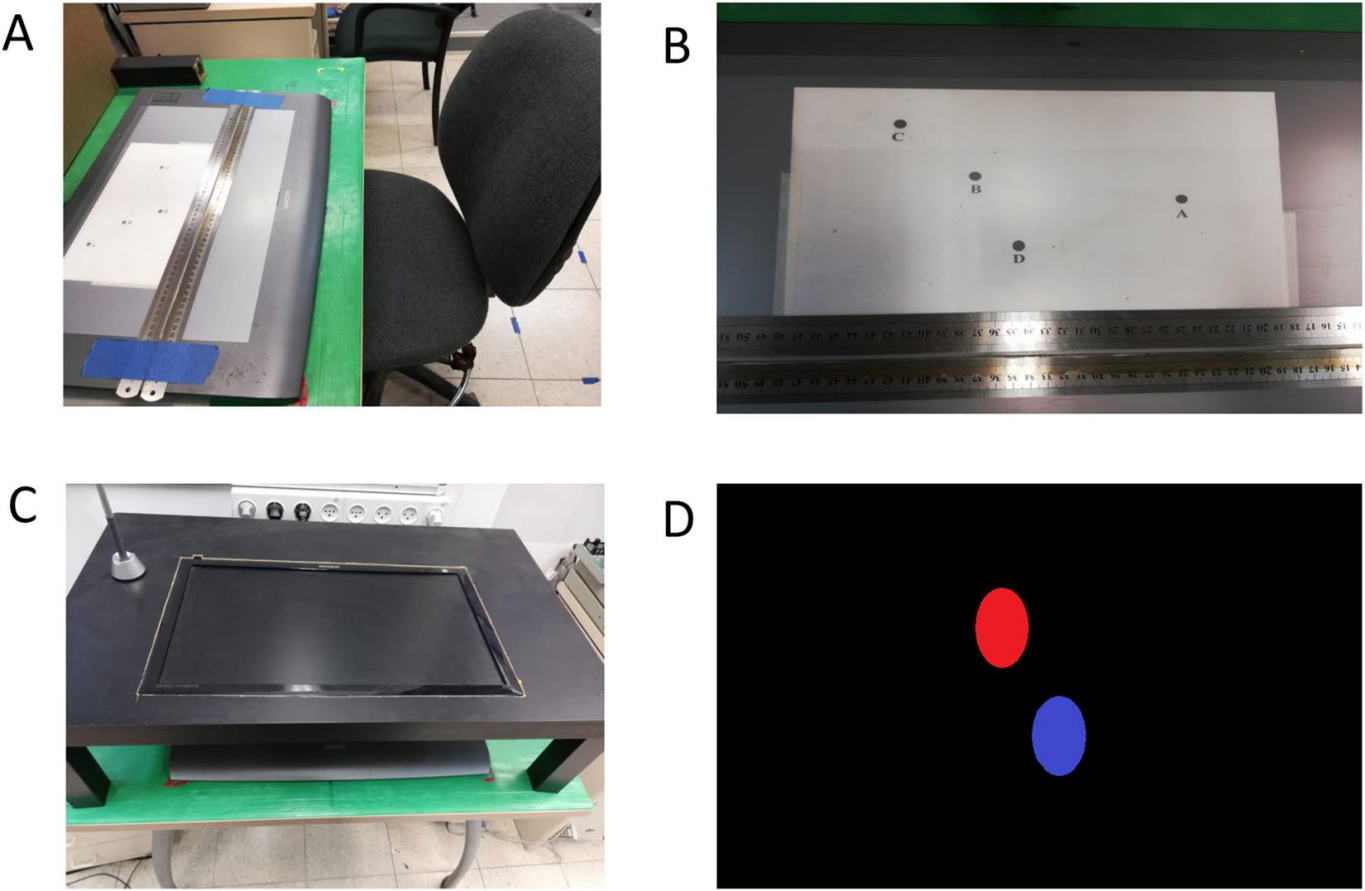
The experimental set-up. (**a**) Participants were seated in front of the tablet at a comfortable distance. (**b**) For the target task, an A4 sheet with the targets was placed on the tablet. Below this, were two rulers that served as a narrow track in which the stylus moved only horizontally during the mirror game. (**c**) A monitor was placed above the tablet during the mirror game, and then removed for the target task. (**d**) In the mirror game, the participants saw on the monitor the red target oval moving left-right and the blue “player” oval which they controlled.

Hand movements were recorded at 200 Hz using a stylus on a graphics tablet (Wacom Intuos3 Platinum 12” x 19”, 5,080 lpi resolution) (Fig. 6(a)). Data collection was carried out using the Repeated Measures software (Friedman)^30^, and the data were analyzed using custom Matlab (MathWorks, Inc.) scripts. The tablet was placed flat on a table at a comfortable distance and participants were seated on an adjustable chair in front of the tablet.

An A4 white sheet of paper was placed on the tablet with 4 targets printed on it (Fig. 6(b)). Accompanying each target (a black dot) was a letter. The movements were recorded but no explicit feedback was given to the participants regarding their path, timing or accuracy, because the stylus did not leave a mark on the tablet.

For the mirror game, the same tablet and stylus were used, along with an additional Samsung computer monitor (29.5 cm × 53.3 cm) placed in a custom-made shelf (made by cutting a hole in the top of an IKEA LACK coffee table and trimming the legs) positioned 20 cm above the tablet (Fig. 6(c)). The monitor was fitted exactly above the tablet so as to give the participants feedback of their placement and movement directly above the location of their hand (Fig. 6(d)). Two metal rulers were taped to the tablet, creating a narrow track. Participants were asked to place the pen in the track and move only left or right within the track.

## Experimental Protocol

### Target task

The experiment protocol was similar to Sosnik *et al*.^16^. Participants drew connecting lines through the 4 dots in a fixed order (A > B > C > D > A) with the stylus. They were instructed to draw the lines without lifting the stylus until they returned to the first target (A) so as to complete a closed shape. Furthermore, they were instructed to connect the dots “as rapidly and accurately as possible” before beginning the experiment. The protocol includes a pre-test phase of 40 trials, followed by 4 training blocks of 30 trials each. Finally, all participants completed an additional post-training block of 40 trials. The experimental groups received the additional instruction of an analogy (“imagine you’re painting with a paint brush and try not to leave paint stains during the drawing”) or explicit instructions (“draw curved lines and go through the targets without a full stop”) after completing the pre-test and again between each training block. Before the post-test, all participants were again instructed to connect the dots “as rapidly and accurately as possible”. The control group received only the original instruction (“connect the dots as rapidly and accurately as possible”), and both control and explicit groups heard their instructions at the same intervals as the analogy group.

### Mirror game

The mirror game is an exercise commonly used in drama and dance classes. In the real-life version of the game, two people attempt to move together in synchrony (forming a mirror image of one another). In many cases, one of the players is designated as the leader and the other is the follower. Our stimuli were adapted from a previous experiment in the lab that recorded subjects playing this game, specifically a participant playing the role of a leader. This game is a case in which players tend to slow down their movements to make it easier to move together. However, they are unable to generate smooth movements when the pace is too slow and may actually find synchronization in faster movements.

The protocol and stimuli for this experiment were taken from Noy *et al*.^18^. Participants were instructed to follow the movements of a red oval moving horizontally on the monitor screen. This was achieved by moving the stylus across the tablet between two metal rulers that restricted vertical movement. Each trial began with the red oval appearing in the center of the monitor screen accompanied by a gong sound. When subjects placed the stylus on the tablet, a blue oval appeared below the red one, representing the “player”. The task included 11 one-minute trials, with breaks between each trial. Each trial contained a unique composition of 2 or 3 frequencies. The first three trials were grouped as a pre-test, and all participants received the same instructions to match the blue oval to the red one as well as they can. After the first three trials, the analogy and explicit groups received further instructions (imagine you are painting with a paint brush or avoid small adjustments accordingly) and were reminded of these instructions every 2 trials thereafter. The control group continued to receive the original instruction in the same intervals as the experimental groups. The trials were randomly assigned for each participant within the pre-test (trials 1–3) and the training phase (trials 4–11).

### Choice of analogy

Following the ideas presented in Poolton, Masters & Maxwell^31^, we constructed an analogy that would be culturally familiar, and we could assume that most participants had either a direct experience with or immediately recognize the reference. Furthermore, we wanted the analogy to invite movement (rather than a static image) and embody movement qualities which we thought would improve performance (in this case smooth as opposed to broken lines). However, we acknowledge this is not the only possible analogy, or even the best one to use in this case. Our choice serves as an example that using an analogy is possible and beneficial. We encourage addressing this issue in future research by developing clear criteria for appropriate analogies, as well as experimental comparison of different analogies.

## Data analysis

### Target task

The data was analyzed using the same techniques described previously in Friedman & Korman^17^. Trials were removed if the stylus movement was not recorded continuously throughout the trial, or participants drew a different pattern within the trial window (such as not completing the shape before the next recording). 2% of individual trials were removed, as well as one participant from the explicit group who did not perform the task as instructed. We filtered the data using smoothing splines, with knots every 6 samples. Plots of mean trajectories and mean tangential velocity were generated by first registering the data^32^. Movement onset was defined as the last time before the first significant peak that the tangential velocity was less than 5% of the peak tangential velocity, and that the stylus pressure was greater than zero. The end of the movement was similarly defined as the last time the tangential velocity was greater than 5% of the peak tangential velocity. We normalized the absolute measures by subtracting the baseline value (taken from the pre-test) from the training and post-test blocks, then dividing by the baseline and multiplying by 100. Spatial error was quantified by measuring the closest distance of the subject’s movements from the edge of each of the specified points, and then summing them. If the subject successfully passed through all the points (i.e., was within the borders of the targets), the spatial error was defined as zero.

Coarticulation was calculated using Matlab software (by Friedman^33^)available online, based on the tangential velocity profile. We defined a coarticulation score as the ratio of the mean of the first and third trough heights to the mean of the peak heights, multiplied by 100. A low coarticulation score (close to zero) indicates that the subject did not coarticulate (i.e. made 4 distinct point-to-point movements), a higher score (closer to 100) indicates greater coarticulation (i.e. did not stop at the intermediate targets and smoothly changed direction). When there were only 3 peaks (due to coarticulation), we used the inflection point (that replaced the peak and trough) for both the missing peak and trough. A similar procedure was performed when there were only two peaks. Normalization of coarticulation scores was performed by subtracting the baseline value. In this case, we did not divide by the baseline value, because many baseline values were close to zero, thus dividing by these values would have produced very large numbers.

### Mirror game

Data analysis was based on methods described in Noy *et al*.^18,34^. Data was removed in cases where participants’ movement was not recorded by the tablet (7% of individual trials had a small portion of the trial removed). We applied a low-pass fourth order two-way Butterworth filter with a cutoff of 5 Hz. Due to the physical constraints of the task, only one dimension of participants movement was used (left-right) and velocities and accelerations were calculated using finite differences. The data was resampled to 100 Hz for the remainder of the analysis. In order to determine participants’ accuracy and overall performance we used the following measures: relative position error (dX), relative velocity error (dV), and mean timing error (dT). Temporal accuracy (dT) was computed as the absolute time difference between zero velocity events in the stimuli and participant data (before registration). dX was calculated by comparing subjects’ positions to the stimulus at the sampling times, after registration. Similarly, dV compares subjects’ velocity to stimulus velocity, after registration. These parameters are defined as follows:

$$dX=\frac{2}{n}\mathop{\sum }\limits_{i=1}^{n}\frac{|{x}_{1}^{i}-{x}_{2}^{i}|}{|{x}_{1}^{i}+{x}_{2}^{i}-2{x}_{c}|}$$

$$dV=\frac{2}{n}\mathop{\sum }\limits_{i=1}^{n}\frac{|{v}_{1}^{i}-{x}_{2}^{i}|}{|{v}_{1}^{i}+{v}_{2}^{i}|}$$

${x}_{1}^{i}$ represents the position of the participant and ${x}_{2}^{i}$ represents the position of the stimulus at time *i* (after registration). Similar notation is used in calculating velocity (*v*). When calculating position error, the location of the center (${x}_{c}$) was subtracted. This is the position that all half sine waves started from.

In order to calculate the jitter, we found the acceleration zero crossings^34^. We only used jitter points that were more than 200 ms (i.e. 5 Hz) apart, as it is unlikely that separate voluntary movements are produced faster than this. We also removed jitter points corresponding to acceleration zero crossings in the stimuli (i.e. peaks and troughs in the velocity). We divided the reciprocal of the time between jitter points by two to get the frequency of a whole wave, which we defined as the jitter frequency. From the distribution of jitter frequencies for a given subject, we calculated the peak jitter frequency based on the maximum value of the kernel density (similar to a smoothed histogram).

## Statistical Analysis

### Target task

We tested the effect of different verbal instructions on coarticulation and movement duration by using mixed design ANOVA tests on the three groups, comparing the training and post-test phases to the pre-test. When these were significant, further post-hoc tests were performed, using Bonferroni-corrected multiple comparisons. As expected, many of the spatial error values were zero, so this measure doesn’t have a normal distribution. In order to compare accuracy between groups throughout the experimental phases we used the Friedman nonparametric statistical test. We also used the Friedman test for analyzing the number of velocity peaks, as this measure did not exceed 4 peaks so also wasn’t normally distributed.

### Mirror game

In light of two missing data values (due to recording error) we used a mixed-effects model (Restricted Maximum Likelihood- REML) to analyze the results in all measures. The test was performed on group means of improvement, calculated by subtracting the baseline measurements in the pre-test from post-test measurements for each subject. Bonferroni-corrected multiple comparisons were applied for post-hoc tests.

All statistical analysis was performed using GraphPad Prism 8.
